# Comprehensive Genomic Analysis and Expression Profiling of Phospholipase C Gene Family during Abiotic Stresses and Development in Rice

**DOI:** 10.1371/journal.pone.0062494

**Published:** 2013-04-30

**Authors:** Amarjeet Singh, Poonam Kanwar, Amita Pandey, Akhilesh K. Tyagi, Sudhir K. Sopory, Sanjay Kapoor, Girdhar K. Pandey

**Affiliations:** 1 Department of Plant Molecular Biology, University of Delhi South Campus, New Delhi, India; 2 National Institute of Plant Genome Research, New Delhi, India; 3 Jawaharlal Nehru University, New Delhi, India; Max Delbrück Center for Molecular Medicine, Germany

## Abstract

**Background:**

Phospholipase C (PLC) is one of the major lipid hydrolysing enzymes, implicated in lipid mediated signaling. PLCs have been found to play a significant role in abiotic stress triggered signaling and developmental processes in various plant species. Genome wide identification and expression analysis have been carried out for this gene family in *Arabidopsis*, yet not much has been accomplished in crop plant rice.

**Methodology/Principal Findings:**

An exhaustive *in-silico* exploration of rice genome using various online databases and tools resulted in the identification of nine PLC encoding genes. Based on sequence, motif and phylogenetic analysis rice PLC gene family could be divided into phosphatidylinositol-specific PLCs (PI-PLCs) and phosphatidylcholine- PLCs (PC-PLC or NPC) classes with four and five members, respectively. A comparative analysis revealed that PLCs are conserved in *Arabidopsis* (dicots) and rice (monocot) at gene structure and protein level but they might have evolved through a separate evolutionary path. Transcript profiling using gene chip microarray and quantitative RT-PCR showed that most of the PLC members expressed significantly and differentially under abiotic stresses (salt, cold and drought) and during various developmental stages with condition/stage specific and overlapping expression. This finding suggested an important role of different rice PLC members in abiotic stress triggered signaling and plant development, which was also supported by the presence of relevant *cis*-regulatory elements in their promoters. Sub-cellular localization of few selected PLC members in *Nicotiana benthamiana* and onion epidermal cells has provided a clue about their site of action and functional behaviour.

**Conclusion/Significance:**

The genome wide identification, structural and expression analysis and knowledge of sub-cellular localization of PLC gene family envisage the functional characterization of these genes in crop plants in near future.

## Introduction

Lipid signaling is one of the major signaling networks triggered in plants as an adaptive response upon exposure to various environmental cues and stress stimuli. Initial impact of a stress stimulus usually happens on the cell membrane and it leads to the hydrolysis of membrane lipids. This process is mediated by phospholipases as various stress stimuli activate phospholipase enzymes, which then catalyse the initial step of phospholipid breakdown and leads to generation of multiple lipid-derived second messengers [1]–[3]. Phospholipase C (PLC) constitutes an important group of lipid hydrolysing enzymes in animals and plants. Two major categories of PLCs have been identified in the plants based on their affinities to different substrates. A well-studied group of phosphatidylinositol-specific PLCs (PI-PLCs), which specifically act upon phosphatidylinositides (PIP_2_) at the membrane and results in the generation of second messengers such as diacylglycerol (DAG) and inositol 1,4,5-trisphosphate (IP_3_). In animals, DAG remains attached to the membrane and it activates protein kinase C (PKC) and IP_3_ is released into the cytoplasm where it bind to the ligand gated Ca^2+^ channel (IP_3_ receptors) in the intracellular membranes and leads to the release of Ca^2+^ from the intracellular reservoirs [4]. However, plant cell lack the equivalents of animal PKC and IP_3_ receptors, therefore the exact function of PLC reaction products DAG and IP_3_ remains enigmatic in plants. Rather, it has been postulated that the phosphorylated products of DAG i.e. phosphatidic acid (PA) and diacylglycerol pyrophosphate (DGPP), and hexakisphosphate (IP_6_), which is the phosphorylated product of IP_3_ may function as second messengers in plants [5]–[10]. Another recently identified class of PLCs in plants is phosphatidylcholine-PLC (PC-PLC), which preferentially hydrolyse phosphatidylcholine (PC) but can also act upon other lipids such as phosphatidylethanolamine (PE) and therefore also referred as non-specific phospholipase C (NPC) [11]–[12]. Both plant and animal PLCs constitute a multigene family and are represented by different isoforms. Mammalian PI-PLC family has been comprised of 13 members, which have been divided into six isoforms β, γ, δ, ε, η and ζ on the basis of their domain structures and organization [13]–[15]. *Arabidopsis* comprised of nine PI-PLCs (AtPLC1-AtPLC9) [16]–[18] and six NPC (NPC1-NPC6) members in its genome [11]. Recently, six PI-PLC members have been characterised in the solanaceae plant tomato [4] and also in other plant species such as potato [19], pea [20], *Physcomitrella* [21], soybean [22] and *Vigna radiata* (Mung bean) [23]. Structurally, a plant PI-PLC is composed of a minimal set of domains including catalytic X domain and Y domain, essential for phosphoesterase activity, followed by Ca^2+^/phospholipid binding C2 domain at the C-terminus and an EF-hand motif like “EF Loop” is present at the N-terminus, however it is poorly conserved across the plant species [15], [18], [19], [24]. Plant PI-PLCs are structurally close to the mammalian PLCζ because both lack a typical Pleckstrin Homology (PH) domain, which is present in other mammalian PLC isoforms [13], [15], [18]. Although structures of several PI-PLCs have been deciphered in plants but knowledge about the structure of NPCs is miniscule. All the six *Arabidopsis* NPCs range 514–538 amino acid in size and comprised of three conserved domains, which are uncharacterised so far [11], [25]. Sequence analysis showed that *Arabidopsis* NPCs bear a high homology with bacterial PC-PLCs [11].

Different PLC members have been implicated in abiotic stress triggered signal transduction in various plant species. All *Arabidopsis* PI-PLC members (except *AtPLC2*) were found to be highly up-regulated under different abiotic stresses such as salinity, drought and cold [18]. Similarly, mung bean PI-PLC member *Vr-PLC3* was readily induced by salinity and drought stresses [23]. Recently, *Arabidopsis* NPC4 was found to be involved in salt stress response in the roots [26]. In addition, some PLC members have also been found to be involved in plant development. The significant role has been suggested for the PI-PLCs in regulating cytosolic calcium level in response to auxin and ABA [10], [15], [27]. Suppression of PI-PLC leads to irregular pollen tube growth and improper plant development [10], [28]. *Arabidopsis* NPC3 and NPC4 have been implicated in the root development [25]. These reports suggest the involvement of PLCs in abiotic stress signaling and development in plant such as *Arabidopsis* and others but the knowledge related to the functions of PLCs is limited in monocot crop plants such as rice. It is expected that PLCs may perform similar function in crop plant rice. Moreover, a comprehensive genome wide account and expression profile of rice PLCs have not been presented till date. The possibility of direct connection between the expression profile and the functional role during abiotic stresses and development, enticed us to perform a genome wide identification and expression analysis of rice phospholipase C gene family.

In this study, we are reporting the entire set of phospholipase C encoding genes in the rice genome for the first time. The rice PLCs have been divided into phosphatidylinositol-PLC (PI-PLC) and phosphatidylcholine-PLC (PC-PLC) or nonspecific PLCs (NPCs, and will be named as NPCs in this manuscript here onwards) based on the domain structure, conserved motif and phylogeny. Detailed expression analysis has been carried out for all the PLCs employing microarray and real time PCR under three abiotic stresses i.e. salt, cold and drought and during various stages of vegetative and reproductive development. In addition, GFP based sub-cellular localization analysis has been performed for a few PLCs to yield a clue regarding their site of action in the cell.

## Results

### Identification and Genomic Distribution of Rice PLCs

**Figure 1:**
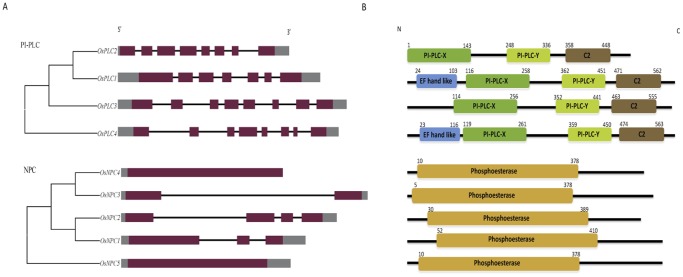
Structural features of rice phospholipase C family. (A) Exon-intron organization is shown for both PI-PLC and NPC groups of rice PLCs and the gene names are mentioned at left. Genes are shown according to their phylogenetic clustering and not in the order of their numbers. Purple bars represent the exon, lines represent the intron while grey colour bars indicate the un-translated region (UTR) both at 5′ and 3′ position. (B) Protein structure of rice PLC gene family is showing the presence of highly conserved characteristic domains PI-PLC-X (Pfam identity- PF00388), PI-PLC-Y (PF00387) and C2 (PF00168) in all the PI-PLC members while EF hand like motif (PF09279) was marked only in two of the members. Phosphoesterase (PF04185) domain is present in all the NPC members. Numbers on each domain depict amino acid residue at the start and end of a particular domain and direction of a protein is depicted by N and C terminal.

Rice genome exploration by various strategies resulted in the identification of a total of nine non-redundant phospholipase C encoding genes. Demarcation of distinct and characteristic domains and sequence motifs by analysis tools such as simple modular architecture research tool (SMART) (http://smart.embl-heidelberg.de/), InterPro (http://www.ebi.ac.uk/Tools/InterProScan) and Pfam (http://www.pfam.sanger.ac.uk/) differentiated entire rice PLC gene family into two major classes, namely phosphatidylinositol specific-PLC (PI-PLCs) and nonspecific phospholipase C (NPC). PI-PLC subfamily is represented by four members, which are named as *OsPLC1* to *OsPLC4* and NPC subfamily is comprised of five members; *OsNPC1* to *OsNPC5*. Gene structure analysis showed that all the PI-PLC genes are made up of multiple intron-exons and intron number varied from six to eight, whereas OsNPCs are simpler in gene structure and members such as *OsNPC4* and *OsNPC5* are intronless while other members have one to three introns (Figure 1A). All the rice PI-PLC genes were found to contain the catalytic PI-PLC-X (PF00388), PI-PLC-Y (PF00387) domain and Ca^2+^/phospholipid binding C2 domain (PF00168), whereas an EF hand like motif (PF09279) was found only in two members OsPLC1 and OsPLC4 (Figure 1B). All the OsNPC members harboured a characteristic catalytic phosphoesterase (PF04185) domain with highly conserved motifs I, II and III (Figure 1B, S1). The protein size of all the PLC members is comparable, with PI-PLCs ranging from 591–599 aa (OsPLC2 is the only exception with 491 aa) and all the NPCs falling in the narrow range of 521–548 aa (Table 1). Expression evidence could be obtained for entire PLC gene family in terms of full length cDNA, total ESTs and microarray profiles (Table 1).

**Table 1:** Various features of PLC genes in rice genome.

RGAP Locus	Gene Name	Full length cDNA	Total ESTs	Protein length(AA)	Intron
LOC_Os07g49330.1	*OsPLC1*	AK061472	104	599	6
LOC_Os03g18010.1	*OsPLC2*	NM_001056327.2	4	491	8
LOC_Os12g37560.1	*OsPLC3*	CT835563	17	591	7
LOC_Os05g03610.1	*OsPLC4*	AK064924	44	599	7
LOC_Os03g61130.1	*OsNPC1*	AK067741	206	546	2
LOC_Os01g72520.1	*OsNPC2*	AK101291	99	521	3
LOC_Os11g38050.1	*OsNPC3*	AK243286	3	536	1
LOC_Os03g63580.1	*OsNPC4*	AC120506.5	1	528	0
LOC_Os01g01190.1	*OsNPC5*	AK101065	19	548	0

### Phylogenetic Analysis

**Figure 2:**
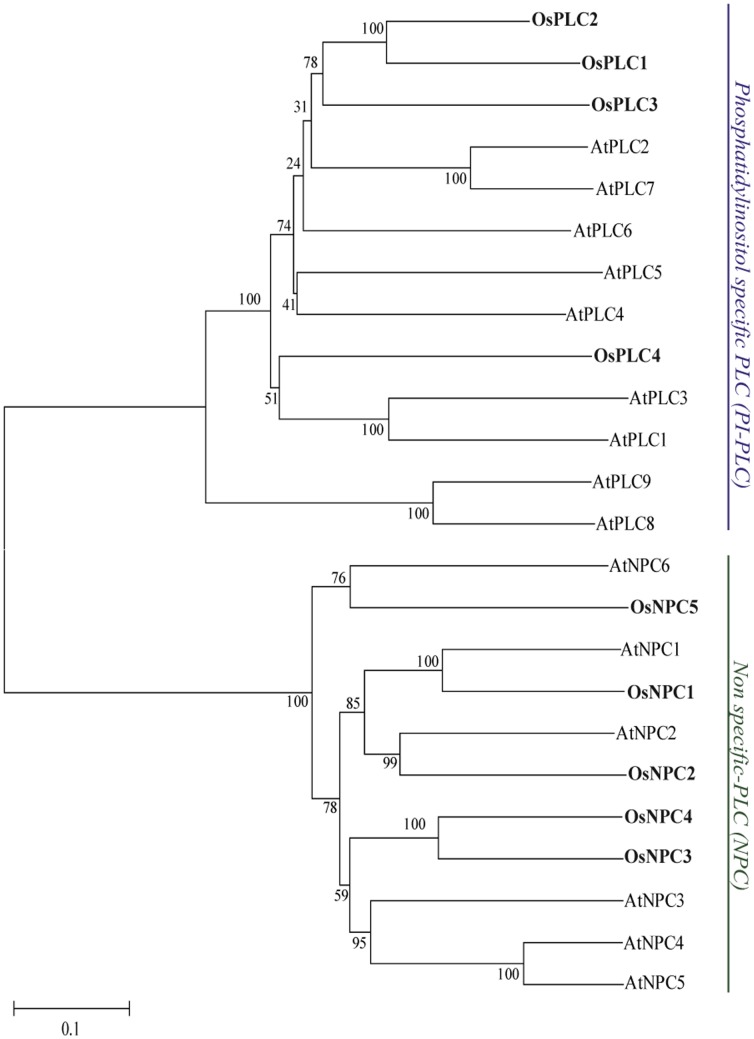
Phylogenetic relationship of rice and ***Arabidopsis*** **PLC gene family.** An un-rooted neighbour joining phylogenetic tree was constructed from the protein sequences of rice and *Arabidopsis* PLCs, including both PI-PLCs and NPCs. Multiple sequence alignment was carried out using clustalX2.0 and the tree was generated using MEGA5. Rice and *Arabidopsis* PI-PLCs and NPCs are clustered together based on significant bootstrap value (>50%). Scale bar indicates 0.1 amino acid substitution per site.

Phylogenetic analysis was performed by taking protein sequences of rice and *Arabidopsis* PLCs to understand the evolutionary relatedness of this gene family in monocot and dicot plant species. Based on the statistical analysis and high bootstrap support value OsPLC1, OsPLC2 and OsPLC3 have very high sequence homology and they make a separate small clade while OsPLC4 falls apart. Moreover, rice PI-PLCs made a phylogenetic clade with *Arabidopsis* PI-PLCs with very low bootstrap support value, suggested their diversification during the course of evolution even after being originated from common ancestor (Figure 2). On the other hand, rice NPCs fall in the same clade with *Arabidopsis* NPCs with very high bootstrap value, indicating a high sequence homology, common ancestory and similar evolutionary path for this group of genes in both the diverse plant species. The phylogenetic analysis helped in the nomenclature and classification of rice PLC genes.

### Expression Analysis under Abiotic Stresses

Expression profile of entire rice PLC gene family was generated using Affymetrix rice genome arrays for the 7 days old rice seedling, treated with three abiotic stress conditions (salt, cold and drought). Most of the members from both PI-PLC and NPC classes were found to be expressed differentially under one or multiple abiotic stresses (Figure S2; Table S1). From the PI-PLC, two members *OsPLC1* and *OsPLC3* were up-regulated while *OsPLC4* was significantly down-regulated under all the three abiotic stresses. *OsPLC2* did not show any significant change in expression under abiotic stresses in comparison to untreated control. Up-regulated PI-PLCs were induced more under salt and drought stresses than under cold stress. Similarly, out of the five rice NPC members four exhibited differential expression pattern while one member *OsNPC3* did not show any significant change in the expression under abiotic stresses. *OsNPC1*, *OsNPC2* and *OsNPC4* were found to be up-regulated while *OsNPC5* exhibited down-regulation. *OsNPC2* and *OsNPC4* were up-regulated in salt and drought stresses together. *OsNPC1* exhibited up-regulation in salt stress and down-regulation under cold stress. *OsNPC5* did not have any significant change under salt stress while commonly down-regulated under cold and drought stresses. None of the PLC gene was expressed commonly under salt and cold or drought and cold stresses together. Under cold stress conditions, none of the PLC genes was found to be up-regulated.

### Expression Analysis during Development

**Figure 3:**
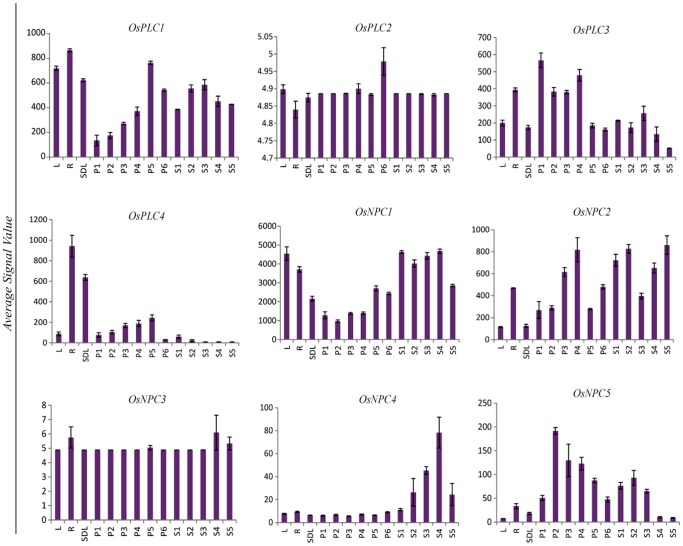
Expression profile of rice PLC gene family during developmental stages. Average signal intensity (GCRMA) value of three replicates from microarray for all the developmental stages (three vegetative; L-mature leaf, R-root, SDL-seven day old seedling, six panicle stages; P1–P6 and five seed stages; S1–S5) has been plotted to show the differential expression. Standard error bars have been shown. Y-axis represents signal values from microarray and X-axis shows different developmental stages.

Affymetrix rice whole genome arrays data was used to generate the expression profile of entire set of rice PLC genes during plant development, covering 11 stages of reproductive development, which includes six stages of panicle (P1–P6) and five stages of seed (S1–S5) development and three vegetative developmental stages (leaf, root and seedling). Most of the PLC genes were found to be expressed in reproductive as well as in vegetative stages of development. A careful in-depth analysis revealed that eight out of nine PLC genes expressed differentially during reproductive developmental stages in comparison to the vegetative developmental stages (Figure 3, S2; Table S2). One of the NPC members, *OsNPC3* did not show any significant variation in expression during developmental stages. From the set of genes, which expressed differentially during reproductive development, five and three genes were up-regulated and down-regulated, respectively. Among the up-regulated genes, two members belonged to PI-PLC (*OsPLC2* and *OsPLC3*) and three genes belonged to NPC (*OsNPC2*, *OsNPC4* and *OsNPC5*). Two genes each were up-regulated and down-regulated commonly during panicle and seed developmental stages. Both the commonly up-regulated genes belonged to NPC group (*OsNPC2* and *OsNPC5*) while both commonly down-regulated genes belonged to PI-PLC group (*OsPLC1* and *OsPLC4*). A typical observation for the unique expression pattern revealed that *OsPLC2* and *OsPLC3* were exclusively up-regulated during panicle developmental stages whereas, *OsNPC4* exhibited exclusive up-regulation during later seed developmental stages. Interestingly, *OsPLC3* which was up-regulated in panicle developmental stages exhibited down-regulation in seed stages while *OsNPC1* showed exclusive down-regulation during panicle developmental stages.

### Overlapping Expression under Abiotic Stresses and Development

Analysis was done for overlapping expression of PLC genes under abiotic stresses and reproductive development, keeping the fact in mind that abiotic stress and reproductive development triggered signaling is mediated by many common factors and at some critical stages these are interconnected phenomenon of plant life cycle [29]–[31]. Remarkably, all the PLC genes which expressed significantly and differentially under abiotic stresses were found to have significant expression pattern during two phases of reproductive development (panicle and seed development) in rice (Table S1, S2). A single NPC member (*OsNPC2*) was found to be up-regulated commonly under abiotic stresses and both panicle and seed development while *OsPLC4* was commonly down-regulated. *OsPLC3* was found to be commonly induced during panicle development and abiotic stresses and *OsNPC4* during seed development and abiotic stresses together whereas, none of the PLCs was down-regulated under these conditions or stages. Observation for overlap between two developmental stages (panicle and seed) revealed that only *OsNPC5* was commonly up-regulated and *OsPLC1* was commonly down-regulated. Interestingly, *OsPLC3*, which was commonly up-regulated in abiotic stress and panicle stages together, was down-regulated in seed developmental stages and similarly, *OsNPC5* was commonly up-regulated in both the developmental phases but was found to be down-regulated under abiotic stresses.

### Microarray Expression Validation under Abiotic Stresses by Q-PCR

**Figure 4:**
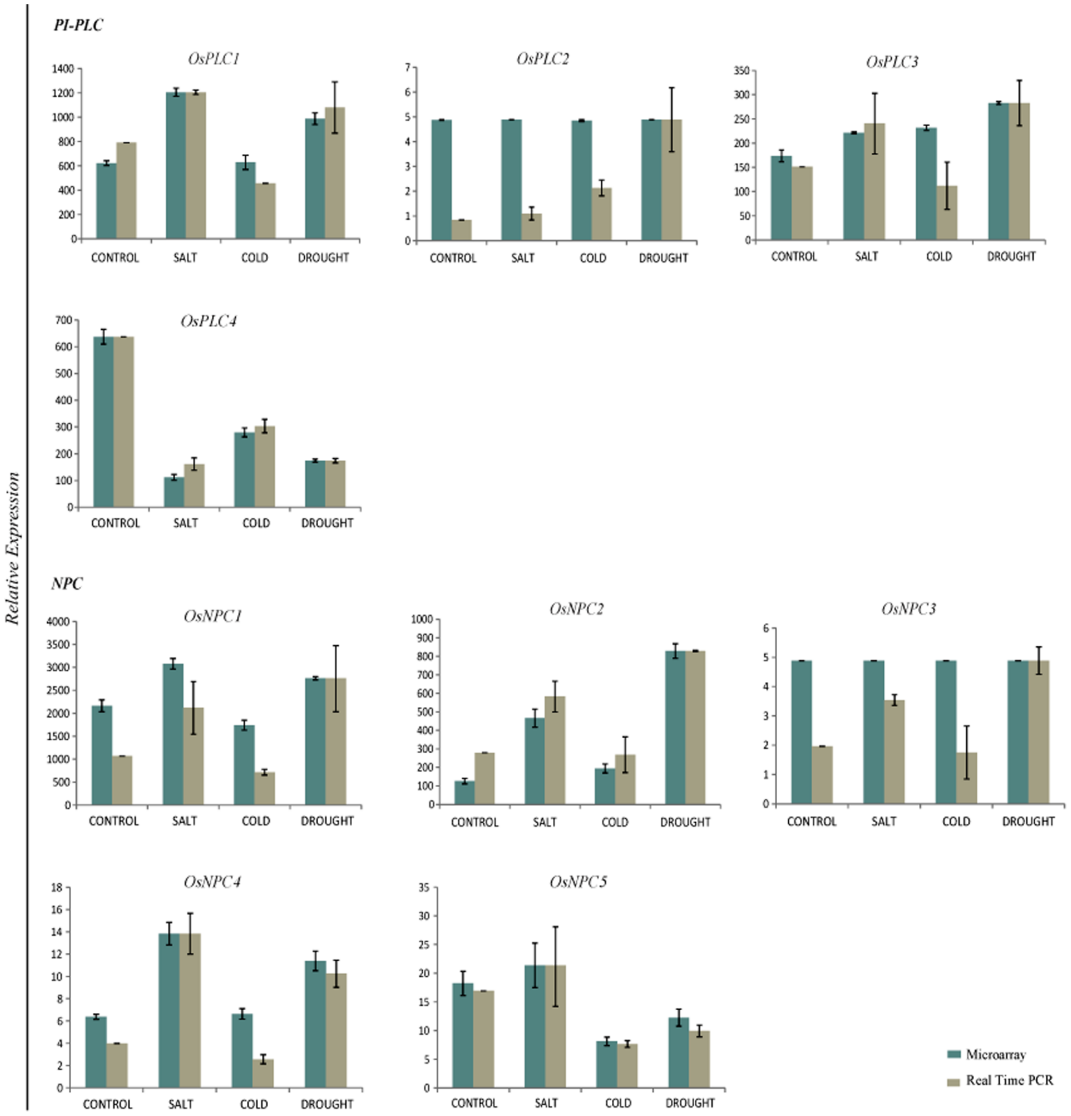
Microarray expression profile validation by quantitative RT-PCR for rice PLC gene family under abiotic stresses. Microarray expression profile was generated using three biological replicate and two biological replicates were used for Q-PCR expression analysis. Standard error bars have been shown for the data from both the techniques. Y-axis represents the normalized expression values and X-axis represents different experimental conditions. Green and grey columns denote the expression values from microarray and real time PCR, respectively.

Quantitative expression analysis was performed by real time RT-PCR for all the rice PLC genes (including both PI-PLC and NPCs) under three abiotic stress conditions (salt, cold and drought) to verify the microarray expression data. Out of a total of nine PLC gene family members, expression pattern for six members was found to be in accordance with microarray expression, however, the magnitude of expression varied slightly in few samples for some genes (Figure 4). For three PLC genes; *OsPLC2*, *OsPLC3* and *OsNPC3* the expression pattern deviated from microarray as shown by the real time PCR analysis. There was no significant differential change observed in the expression of *OsPLC2* and *OsNPC3* by microarray under the treatment of abiotic stresses w.r.t. control whereas, real time PCR revealed up-regulation of *OsPLC2* under cold and drought conditions and *OsNPC3* up-regulated under salt and drought stresses. Since actual transcript level of these two genes is very low in all the conditions as suggested by both the techniques, even minute differences in the sensitivity to detect transcripts might account for the deviation in the relative expression profile. Similarly, *OsPLC3* was shown to be down-regulated under cold stress condition in contrary to the slight up-regulation revealed by microarray. This kind of discrepancy in the expression pattern by these two techniques has been reported in several previous studies also [30]–[32].

### Cis-regulatory Elements in the Promoter of rice PLC Genes

**Figure 5:**
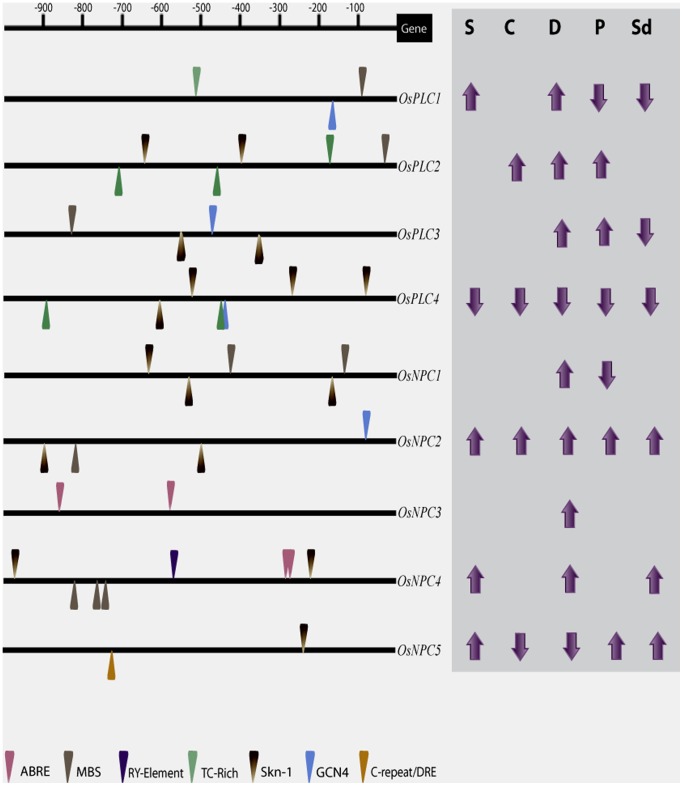
Promoter analysis of rice PLC gene family. *Cis*-regulatory elements in the 1 kb upstream region (from translation start site) of the promoter of all the rice PLC genes were analysed using PlantCARE database. Various elements such as ABRE, MBS, TC-rich repeat, C-repeat/DRE, RY-elements, Skn-1 and GCN4 are present in the forward and reverse strands and are indicated by the arrowhead mark above and below the line, respectively. Scale over the promoters indicates the specific location of the elements. Differential regulation (up- or down-regulation) of the genes under different abiotic stresses; S-salt, C-cold, D-drought and during reproductive developmental phases; P- panicle and Sd- seed, is indicated on the right side of each gene.

*In-silico* analysis of 1 kb upstream region (from translation start site) of all the rice PLC members revealed the presence of various regulatory elements, which included ABRE (abscisic acid responsive element), MBS (myb binding site), Skn-1, RY-element, GCN4, TC-rich repeat and C-repeat/DRE (Figure 5). These motifs have been previously known to regulate various stress responses and plant development [33]–[37]. The motifs were variably present in the promoter of all the PLC genes. Most regulatory elements were present on the 1 kb upstream region of *OsNPC4* containing eight elements including three MBS and two ABRE motifs, which might account for the responsive nature of this gene under abiotic stresses and seed developmental stages. Surprisingly, the promoter of *OsPLC4*, which has been down-regulated under all the abiotic stresses and developmental stages, contain seven *cis*-regulatory elements. All the rice PLC genes contained at least two regulatory elements in their promoter region.

### Sub-cellular Localization of PLC Proteins

**Figure 6:**
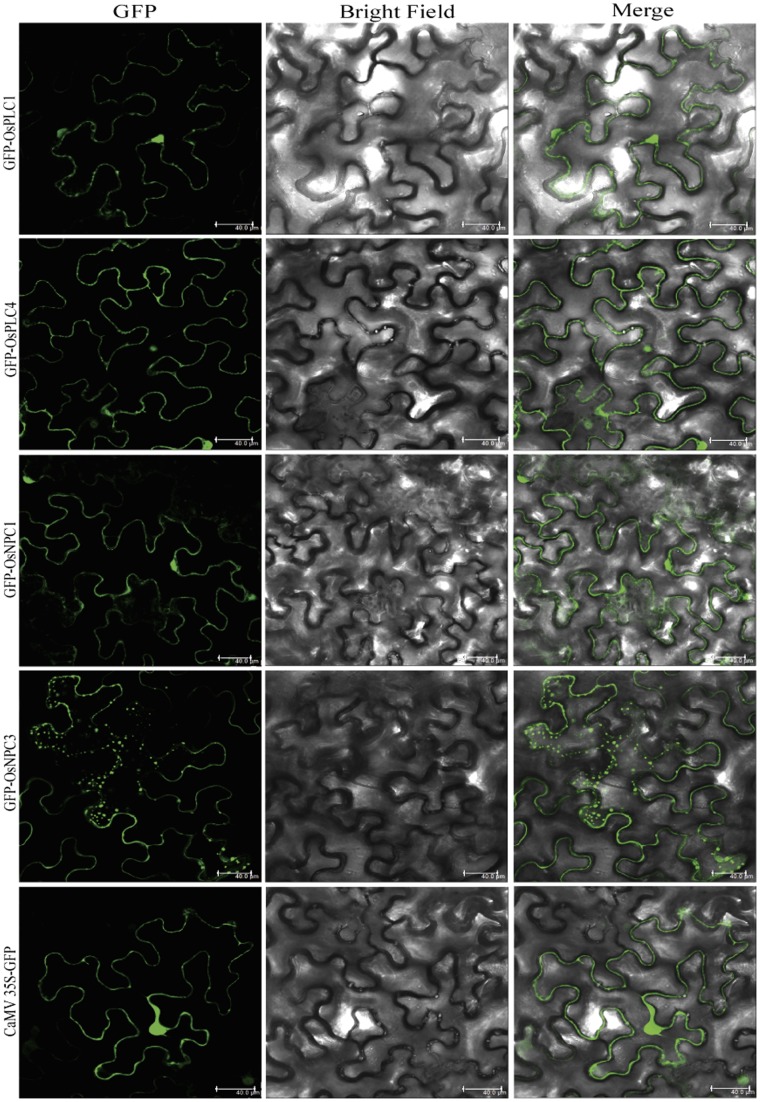
Subcellular localization of OsPLC proteins in ***Nicotiana benthamiana*** **cells.** Agrobacterium-infiltrated tobacco leaves expressing the GFP-PLC fusion protein driven by the 2XCaMV 35S promoter. Confocal images of fluorescence (green) for cell expressing OsPLC1 and OsPLC4 are showing their distribution throughout the cytoplasm and nucleus (first and second row). Expressed GFP-OsNPC1 fusion protein, mainly localized to cytoplasm and small spots like structures in the cell (third row), and GFP-OsNPC3 fusion protein showed preferential localization to small punctate structures in the cell (fourth row). Cells transformed with vector only (CaMV35S-GFP) are shown in the lowermost row. All the images were taken in 5 different sections in z direction and merges together. Scale bar = 40 µm.

**Figure 7:**
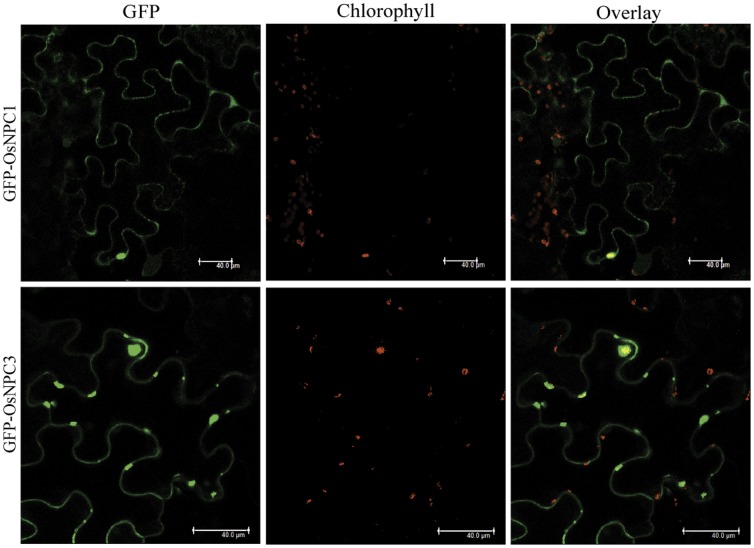
Co-localization of rice NPC proteins with chlorophyll auto-fluorescence of the cell. Confocal images of *Nicotiana benthamiana* cell expressing GFP-OsNPC1 (upper panel) and GFP-OsNPC3 (lower panel). Green GFP signal merges with red auto-fluorescence of chloroplast, as seen in the merged regions (yellowish orange) in the overlay. All the images were taken in 5 different sections in z direction and merges together. Scale bar = 40 µm.

To assess the functionality of rice PLC genes, two representative members from both PLC classes; PI-PLC (OsPLC1 and OsPLC4) and NPC (OsNPC1 and OsNPC3) were selected for subcellular localization analysis. All four genes were fused to C terminus of GFP as the reporter molecule, which were transiently expressed in both *Nicotiana benthamiana* and onion epidermal cells. OsPLC1 and OsPLC4 were found to be distributed throughout the cytoplasm as well as in the nucleus of *Nicotiana* cells (Figure 6) and similar localization pattern was observed in onion epidermal cells (Figure S3). Whereas, in the case of OsNPC1 and OsNPC3 dotted structure patterns were observed, which were found to be overlapping with the red chlorophyll auto-fluorescence as seen in the overlay with the green fluorescence (Figure 7). This suggested that OsNPC3 protein might be localized in the chloroplast/plastids. OsNPC1 appears to be localized mainly in the cytoplasm; however, the closer look in the magnified image of the cell suggested that this protein is concentrated around the chloroplast as it was surrounding by a patch of chloroplasts, which were clearly visible with chlorophyll auto-fluorescence (Figure S4).

## Discussion

### Phospholipase C Gene Family in Rice

Exhaustive genome wide exploration through various databases and tools revealed the existence of a total of nine phospholipase C encoding genes in rice genome. Entire rice PLC gene family could be differentiated into two major classes namely; PI-PLC and NPC with four and five members, respectively, based on in-depth sequence, structure and phylogenetic analysis. PI-PLC represent a major class of PLC in plants as suggested by the genomic survey of some of the model plants such as *Arabidopsis,* which has nine PI-PLC member in its genome [16]–[18], and tomato (*Lycopersicon esculentum)* genome encodes six PI-PLC genes [4]. Surprisingly, rice genome found to have fewer PI-PLC encoding genes in comparison to *Arabidopsis,* which has much smaller genome size. In rice genome, we could identify five members of NPC (*OsNPC1*-*5*) class, which is recently discovered class of plant PLCs and till date it has been reported in the genome of *Arabidopsis* only with six members [11]. Domain and motif analysis revealed the presence of characteristic PI-PLC-X and PI-PLC-Y catalytic domains and phospholipid binding C2 domain at the C-terminal in all the PI-PLC members. Notably, an EF-hand like motif was found only in two PI-PLC members (OsPLC1 and OsPLC4). Previously, it has been suggested that EF-hand motifs in plant PI-PLCs may interact with the C2 domain and facilitate the membrane targeting and catalytic activity [15], [16]. Similarly, all the NPC members were identified by the presence of highly conserved signature motif I, II and III (Figure 1B, S1), which confirmed the integrity of all the identified rice PLC genes. Structural analysis of rice PLC genes in terms of intron-exon architecture showed that all the PI-PLC members contain 6–8 introns and this intron-exon pattern is conserved among *Arabidopsis* PI-PLC members also. However, NPC members of rice contain 0–3 introns while *Arabidopsis* genes belonging to this group of PLC are comprised of 2–4 introns. Moreover, *Arabidopsis* NPC members are divided into two groups (I and II) based on the number of introns they contain. Gene members of *Arabidopsis* group I of NPC found to contain 2 introns while all the members of group II contain 4 introns [25]. This gene structure pattern could not be observed in rice NPC genes; therefore, they could not be divided into groups like *Arabidopsis*. Analysis of protein size of rice PLCs showed that all the PI-PLC proteins are ∼66 kDa while NPCs are ∼60 kDa and approximately similar protein size was noted for their *Arabidopsis* orthologs. This indicated towards the structural conservation of PLC proteins across different plant species.

### PLCs are Evolutionarily Diverse in Rice and Arabidopsis

Evolutionary study of rice PLCs members indicated that three members of PI-PLCs (OsPLC1, OsPLC2 and OsPLC3) group together with highly significant bootstrap support value in a phylogenetic tree and OsPLC4 is placed separately (Figure 2). Notably, these three members have similar expression profile under abiotic stresses and development with up-regulation in these conditions/stages while OsPLC4 showed completely opposite behaviour i.e. down-regulation in all the conditions and stages. This observation indicates towards the functional significance of phylogenetic closeness of the rice PI-PLC members. In-depth analysis of the phylogeny showed that most of the rice PI-PLCs are separated from their *Arabidopsis* orthologs and seems to have gone through a diverse evolutionary path even after being originated from a common ancestor. Interestingly, NPC members from both the plant species seem to be evolutionarily more closed and conserved at sequence level. Moreover, gene duplication analysis showed that rice PLC genes did not participate in significant duplication event. In contrary, their *Arabidopsis* counterparts have gone through substantial gene duplication process and members from PI-PLC and NPC classes have been found in the clusters of tandem duplicates on respective chromosomes (Table S3). This pattern of duplication suggested that PLC gene family in *Arabidopsis* have evolved through gene duplication process while rice PLC gene family has followed a diverse path rather than gene duplication event.

### PLCs are Vital Players in Abiotic Stress Triggered Signaling and Plant Development

To get a clue about the functional role of various members of rice PLC gene family, genome wide expression profile was generated for entire PLC members using gene chip microarray data. The analysis showed that most of the members from PI-PLC and NPC groups were differentially expressed under three abiotic stresses (salt, cold and drought) in the 7 days old rice seedlings. From this microarray analysis, a higher number of genes were found to be up-regulated than down-regulated under one or more stress conditions. This expression pattern was also validated by real time RT-PCR, which corroborated the expression pattern for most of the genes and conditions. This critical information about the expression status of rice PLC genes suggested that members from this group of phospholipases might play a significant role in abiotic stress triggered signaling and stress tolerance in crop plants. This assumption has been supported by the previous studies in different plant species where this group of genes has been implicated in abiotic stress mediated signaling and tolerance. In *Arabidopsis*, RT-PCR based expression analysis showed that almost all the PI-PLC members (except *AtPLC2*) were induced several fold under similar abiotic stresses and ABA treatment [17]–[18]. Similarly, expression was rapidly induced for one of the mung bean (*Vigna radiata* L.) PI-PLC members, *Vr-PLC3* under drought and high salinity conditions [23]. Wang and co-workers showed that overexpression of a maize PLC1 (*ZmPLC1*) could lead to better drought tolerance [38]. Recently, one of the tobacco PLC genes has been characterized where its transcript levels were escalated under salt and drought stresses and the transgenic plants overexpressing this gene could tolerate these abiotic stresses [39]. Recently identified group of plant PLCs, NPC has been mainly implicated in phosphate starvation; however, some recent reports have shown their significant role in abiotic stress signaling. *Arabidopsis* plants deficient in NPC4 were found to have reduced tolerance to salt and dehydration stresses while overexpression plants exhibited improved tolerance to these stresses [12]. Similar results were also found for the NPC4 in *Arabidopsis* roots in another recent study [26]. Moreover, the promoters for entire set of rice PLCs were found to contain one or more important *cis*-regulatory elements such as ABRE, MBS, TC-rich repeat, C-repeat/DRE (Figure 5), which might also be involved in regulation of expression of rice PLCs under these stress conditions, since these elements have been known to regulate abiotic stress mediated responses of various genes. Apart from PLCs, other important classes of rice phospholipases (PLA and PLD) have also been found to be expressed differentially under similar abiotic stress treatments [40]–[42], which signify the role of different classes of phospholipases and lipid mediated signaling in abiotic stress physiology and responses in crop plants.

Apart from understanding the effect of different stresses on the transcript levels of various rice PLC genes, it is also important to conceive about the expression level of this group of genes at various stages of development and also relate the effect on abiotic stresses and plant development. Moreover, genes involved in regulation of panicle and seed development are major determinants of yield and productivity of rice crop. Therefore, it is of great significance to understand their regulation, which will be an important step towards developing high yielding rice varieties. Relevant to this, we found that eight out of a total of nine rice PLC genes were differentially expressed during the critical stages of rice development including six panicle (P1–P6) and five seed stages (S1–S5). These critical stages represent the floral organ development (P1), meiosis (P2–P3) to mature pollen (P6) and early globular embryo (S1), embryo morphogenesis and maturation (S3–S4) to dormancy and dehydration tolerance (S5) phase of reproductive development. Different members from both PI-PLC and NPC have been found to be significantly up-regulated and down-regulated during these stages either specifically or commonly (Figure 3, S2). Spatial and temporal fluctuations in the expression of these genes might affect specific developmental stages either positively or negatively and ultimately might affect overall development and yield of rice grains. Abiotic stresses are among the major factors, which significantly influence the growth, development and productivity of the plants in the environment and at the molecular level abiotic stresses (especially drought) and plant development has been found to be interconnected in previous studies [29]–[31]. Keeping this view in mind, we have investigated the PLC genes with overlapping expression in abiotic stresses and developmental stages. Surprisingly, all the genes, which expressed differentially under abiotic stresses, were also involved in one or both the developmental phases (panicle and seed development stages). The information pertaining to the expression of such genes (*OsPLC3*, *OsPLC4*, *OsNPC2* and *OsNPC4*) might explain for a connection or interaction of signaling pathways triggered by abiotic stresses and during development resulting in the “cross talk” of these signaling cascades. This cross talk could possibly be the result of involvement of some common signaling components such as “calcium” or “ABA” which have been implicated at the conjuncture of abiotic stress and developmental triggered responses [43]–[47]. This speculation might be justified by the presence of ABRE element in the promoter of gene such as *OsNPC4,* which is commonly induced during abiotic stresses and seed development. Furthermore, overexpression of *Brassica napus PI-PLC2* resulted in alteration in expression level of genes, which included stress related genes, hormone regulated and regulatory genes, and a number of calcium regulated genes and transcription factors. Moreover, *Brassica napus PI-PLC2* overexpressing transgenic plants exhibited improved drought tolerance and early flowering [48]. NPC class of plant PLCs has not been investigated for its functional role sufficiently, but recently significant role has been established for *Arabidopsis* NPC3 in root development [25].

### Subcellular Localization of OsPLCs Revealed Possible Site of Action

Knowledge obtained about a gene from genomic analysis and its expression status at transcript level require functional validation at protein level for further in-depth analysis and characterization. Therefore, we successfully assessed the subcellular localization of four rice PLCs in *Nicotiana benthamiana* as well as in onion epidermal cells. Both the members of PI-PLC class; OsPLC1 and OsPLC4 were clearly found to be localized to cytosol and nucleus. In higher plants, PI-PLCs have been divided into two groups as one group found to be present predominantly in cytosol and other group at plasma membrane [16], [22]–[23], [49]–[50]. However, a recent study revealed that *Arabidopsis* and rice PLCs might be localized in the cytoplasm, nucleus and the mitochondria, and lack a transmembrane domain, myristoylation and GPI-anchor protein modifications [51]. Sub-cellular localization analysis of two members of rice non-specific PLCs; OsNPC1 and OsNPC3 suggested the possible localization of OsNPC3 to the chloroplast in *Nicotiana benthamiana*, as the GFP-OsNPC3 fluorescence could be co-localized with the chlorophyll auto-fluorescence (Figure 7). On the other hand, OsNPC1 seems to be a cytosolic protein localized in close proximity to chloroplast (Figure S4). Non-specific PLC is the recently identified class of plant PLCs, therefore, not much has been known about their sub-cellular localization and functional aspects, however, in *Arabidopsis* one of the NPC member NPC5 is cytosolic and found to be translocated to chloroplast under phosphate starvation condition, and the DAG produced from the activity of NPC5 is used in galactolipid biosynthesis [11], [52]. Therefore, rice NPC members might also be participating in similar cellular processes. The diverse localization pattern for rice PLC proteins indicates their significant role in important cellular processes regulated by lipid mediated signaling.

### Conclusions

In conclusion, in this study, for the first time we are presenting a comprehensive account of the entire set of PLC encoding genes in the rice genome by elaborating their genomic organization, sequence and phylogenetic analysis. Detailed expression analysis proposed a vital role for PLC enzymes in abiotic stress and development triggered lipid signaling in rice. Successful sub-cellular localization of selected PLC members provide a clue about their possible site of action and involvement in concerned processes in the plant cell. This study opens up the avenues for the detail functional characterization of candidate PLC genes to establish their certain role in lipid mediated signaling in crop plant rice.

## Materials and Methods

### Identification of PLCs in the Rice Genome

Rice genome databases in RGAP version 6.1 and NCBI were explored to identify the putative PLC members, using different keywords such as “phospholipase C”, “phosphatidylinositol-phospholipase C”, “phosphatidylcholine - phospholipase C” and “phosphoesterase” and by phospholipase C domain annotation in SUPERFAMILY database (http://supfam.cs.bris.ac.uk/SUPERFAMILY). Hidden Markov Model (HMM) profiles generated by the seed alignment at default parameters (E value –1.0) were obtained for different PLC classes from Pfam database and used to search protein database at RGAP. *Arabidopsis* PLC sequences were searched and obtained from TAIR 10.0 (The Arabidopsis Information Resource). Homology search was performed using *Arabidopsis* sequences as query, employing BLASTP and BLASTN tools both in RGAP and NCBI. The putative rice PLC sequences having very high homology with *Arabidopsis* PLCs were retained for further analysis. After the merger of results from all these strategies, unique entries (with unique locus ID) were identified to remove the redundancy. All the sequences were further scanned through SMART, InterPro and Pfam tools for the presence of characteristic and functionally important domains and motifs.

### Multiple Sequence Alignment and Phylogenetic Analysis

To mark the conserved and characteristic catalytic domains and motifs, the amino acid sequences of all the rice PI-PLCs and NPCs were aligned using ClustalW method in MegAlign software of DNASTAR. For phylogenetic analysis, the protein sequences of all the non-redundant PLC candidates from rice and *Arabidopsis* were used for multiple sequence alignment employing clustalX (version 2.0). The multiple sequence alignment was used to construct a maximum likelihood, un-rooted phylogenetic tree to infer the evolutionary history for all the PLC genes. The tree was generated by neighbour-joining (NJ) algorithm with p-distance method and pairwise deletion of gaps, employing MEGA version 5 [53], using default parameters. A bootstrap statistical analysis was performed with 1000 replicates to test the phylogeny.

### Gene Nomenclature and Properties

Rice PLC genes (PI-PLC and NPCs) have been named according to their *Arabidopsis* orthologs as suggested by phylogenetic analysis and by their sequence and domain structure analysis. Various properties and attributes of PLC genes such as locus ID, protein size (AA), introns and expression evidence in terms of total number of ESTs were extracted from RGAP 6.1 and full length cDNA accession from knowledge based oryza molecular biological encyclopedia (KOME) (http://cdna01.dna.affrc.go.jp/cDNA) and NCBI.

### Plant Growth and Abiotic Stress Treatment

The tissues were collected for different stages of panicle (P1–P6) and seed (S1–S5) development from field grown rice plants (*Oryza sativa* ssp. *Indica* var. IR64) and instantly frozen in liquid nitrogen to avoid any wounding or damage during handling. As per the requirement of this journal, we declare that no specific permits were required, the location is not privately owned and IR64 is not endangered or protected species of rice.

For stress treatment, dehusked rice seeds were sterilized with 70% ethanol and 0.1% HgCl_2_ and grown in culture room at 28±1°C with a daily photoperiodic cycle of 14 h light/10 h dark. After 7 days growth, salt, cold and drought stress treatments were given to the seedlings parallel to untreated control according to Ray et al., 2007 [54]. Treated seedlings were immediately frozen in liquid nitrogen.

### Gene Chip Microarray Experiments

Microarray experiments were performed taking 11 reproductive stages (P1–P6 and S1–S5; representing panicle and seed developmental stages, respectively) of rice along-with three vegetative stages (mature leaf, 7 days old seedling and their roots) and three abiotic stress conditions, i.e. cold, salt, and drought. For each stage/treatment, total RNA was isolated from three biological replicates and microarray experiments were carried out using 51 Affymetrix Gene Chip Rice Genome Arrays (Gene Expression Omnibus, GEO, platform accession number GPL2025). For further detailed analysis of the data, the raw data (*.cel) files, generated from all the chips were imported to Array Assist 5.5.1 software (Stratagene, USA). Microarray analysis was performed according to Ray et al., 2007 [54]. The microarray data generated from this study has been deposited in the gene expression omnibus (GEO) database at NCBI, under the series accession numbers GSE6893 and GSE6901.

### Expression Analysis by Real Time RT-PCR

Real time RT-PCR was performed to validate the microarray data for all the rice PLC genes under abiotic stresses. The primers were design for all the genes preferentially from 3′ end, using PRIMER EXPRESS (PE Applied Biosystems, USA), with default parameters. Primers were checked for their specificity using BLAST tool of NCBI and dissociation curve analysis after the PCR reaction (Table S4). First strand cDNA was prepared from 4 µg of DNase treated total RNA, in 100 µl of reaction volume using high-capacity cDNA Archive kit (Applied Biosystems, USA). KAPA SYBR FAST Master Mix (KAPABIOSYSTEMS, USA) was used to detect the expression for the genes in ABI Prism 7000 sequence detection system (Applied Biosystems, USA). Biological duplicates of each sample were taken for the analysis. The average Ct values were calculated by taking the average of three technical replicates for each sample. The cDNA variance among samples was normalized using *ACTIN*, as the endogenous control. Relative expression values were calculated by ΔΔCt method and normalized the data against the maximum average expression value from microarray.

### Promoter Analysis

One kb upstream region from the translation start site was extracted from RGAP version 6.1 for all the rice PLC genes to identify various *cis*-acting regulatory elements in their promoter. One kb upstream region was subsequently analysed in plantCARE [55] database and various *cis*-regulatory elements and motifs involved in abiotic stress responses and development were identified.

### GFP-PLC Constructs Preparation for Sub-cellular Localization

Protein coding region (without stop codon) of respective PLCs were amplified from stress treated cDNA of rice (IR64) with gene specific primers (Table S5), using iProof high-fidelity DNA polymerase (Bio-Rad). All the GFP-PLC constructs were prepared by cloning the amplified PLC CDS in Gateway® entry vector pENTR-D/TOPO (Invitrogen) and subsequent mobilization to gateway compatible binary vector pSITE2CA [56] by Gateway cloning protocol (Invitrogen). In pSITE2CA vector, the 2XCaMV 35S promoter regulates the expression of cloned gene. Successful preparation of all the constructs was confirmed by sequencing.

### Nicotiana Benthamiana Infiltration, Particle Bombardment in Onion Peel and Confocal Microscopy

*Agrobacterium tumefaciens* (GV3101::pMP90) was transformed with GFP-PLC construct plasmid, which was then used to transfect the leaves of 4–6 weeks glass-house grown *Nicotiana benthamiana* plants. After infiltration, plants were kept for incubation (3–5 days) under 12 h light/12 h dark photoperiodic cycle at 20°C with 60% atmospheric humidity [57]–[58]. Biolistic or particle bombardment in onion epidermal peels was carried out according to Singh et al., 2012 [41]. Transiently transformed epidermal peel cells were analyzed in TCS SP5 laser scanning confocal microscope (Leica, Germany) to detect the fluorescence. GFP fluorescence signals were detected at 500–535 nm after excitation at 488 nm. Auto-fluorescence of plastids was detected at 650 to 720 nm. For the co-localization experiments, sequential scanning was done for both the channels and then merged together to shows overlapping signals. All the images were further processed using Leica LAS AF Lite software.

## Supporting Information

Figure S1 **Multiple sequence alignment of rice PI-PLC and NPC showing the consensus and conserved domains and motifs.** Protein sequences were aligned for both the PLC classes separately employing clustalW tool of MegAlign-DNA STAR. The conserved and characteristic domains and sequences of PI-PLCs and NPCs have been marked with red underline.
(TIF)

Figure S2 **Microarray expression profile of rice PLC gene family.** Separate heatmaps are depicting the expression profiles of PI-PLC and NPC groups of rice PLC under abiotic stresses and development. Three abiotic stress conditions are denoted by C: cold, D: drought, S: salt and SL: seven day old untreated seedling as control. Developmental stages include three vegetative stages L: mature leaf, R: root, SL: seven day old seedling and eleven reproductive stages comprising six panicle developmental stages [P1 (0–3 cm), P2 (3–5 cm), P3 (5–10 cm), P4 (10–15 cm), P5 (15–22 cm), and P6(22–30 cm)] and five stages of seed [S1 (0–2 DAP), S2 (3–4 DAP), S3 (4–10 DAP), S4 (11–20 DAP) and S5 (21–29 DAP)]. The colour scale at the bottom of each heat map is given in log2 intensity value.
(TIF)

Figure S3 **Subcellular localization of rice PLC proteins in onion epidermal cells.** Onion epidermal cells expressing the GFP-PLC fusion protein driven by the 2X CaMV35S promoter. Confocal images of fluorescence (green) are shown for onion cell expressing GFP-OsPLC1 and GFP-OsPLC4 fusion protein showing its distribution throughout the cytoplasm and nucleus (upper two rows); expressed GFP-OsNPC1 and GFP-OsNPC3 fusion protein showing their preferential dotted localization (third and fourth rows). Cells transformed with vector only (CaMV35S-GFP) are shown in the lowermost row. All the images were taken in 5 different sections in z direction and merges together. Scale bar = 40 µm.
(TIF)

Figure S4 **Preferential localization of OsNPC1 proteins in close proximity of chloroplast in** ***Nicotiana*** **cells.** Expressed GFP-OsNPC1 fusion protein in *Nicotiana* cells, the upper panel showing cytoplasmic localization with small spots in the cell, which surround the chloroplasts in the overlay (Scale bar = 40 µm), which can be seen clearly in the magnified view of the spot in the lower panel. (Scale bar = 10 µm). All the images were taken in 5 different sections in z direction and merges together.
(TIF)

Table S1 **Microarray expression data for OsPLCs under three abiotic stresses.**
(XLSX)

Table S2 **Microarray expression data for OsPLCs during development.**
(XLSX)

Table S3 **Gene duplication exhibited by** ***Arabidopsis*** **PLC gene family.**
(XLSX)

Table S4 **List of primers used for real time RT-PCR expression analysis of OsPLCs.**
(XLSX)

Table S5 **List of primers used for cloning of OsPLCs for sub-cellular localization.**
(XLSX)
