# Supplementary figures and images for: Comprehensive Genomic Analysis and Expression Profiling of Phospholipase C Gene Family during Abiotic Stresses and Development in Rice
Source: PLoS One. 2013 Apr 30;8(4):e62494. doi: 10.1371/journal.pone.0062494 (PMC3640072; doi:10.1371/journal.pone.0062494)

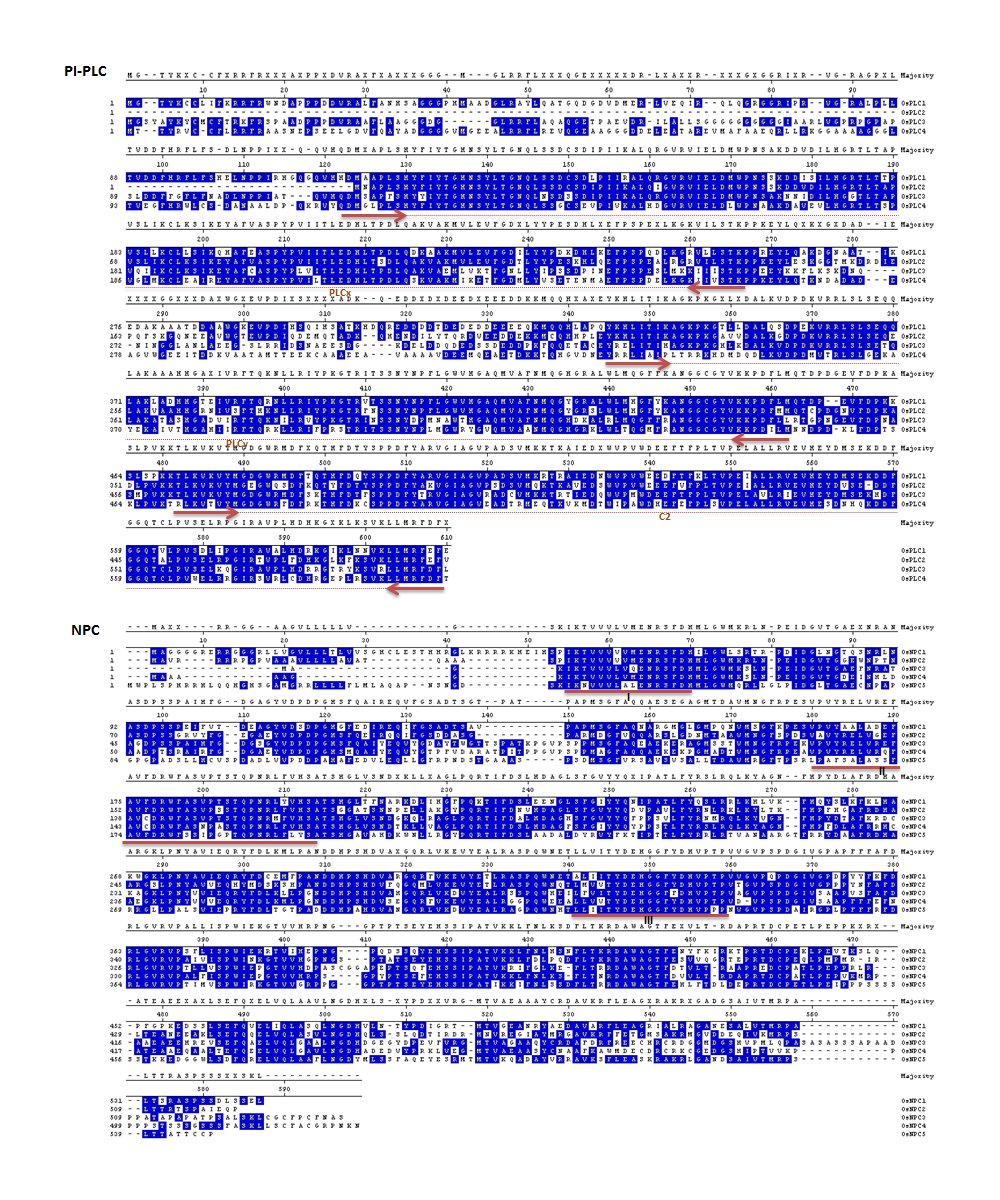

Supplement: Figure S1 — Multiple sequence alignment of rice PI-PLC and NPC showing the consensus and conserved domains and motifs. Protein sequences were aligned for both the PLC classes separately employing clustalW tool of MegAlign-DNA STAR. The conserved and characteristic domains and sequences of PI-PLCs and NPCs have been marked with red underline. (TIF) [file pone.0062494.s001.tif]

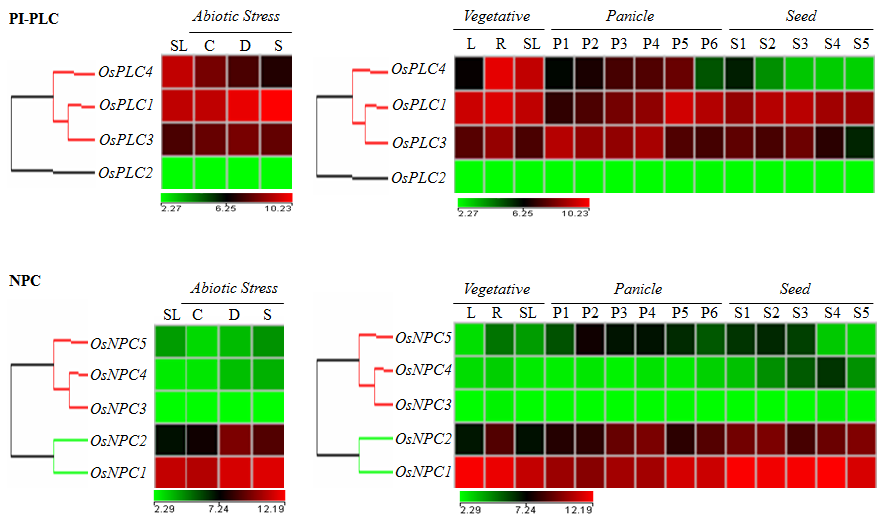

Supplement: Figure S2 — Microarray expression profile of rice PLC gene family. Separate heatmaps are depicting the expression profiles of PI-PLC and NPC groups of rice PLC under abiotic stresses and development. Three abiotic stress conditions are denoted by C: cold, D: drought, S: salt and SL: seven day old untreated seedling as control. Developmental stages include three vegetative stages L: mature leaf, R: root, SL: seven day old seedling and eleven reproductive stages comprising six panicle developmental stages [P1 (0–3 cm), P2 (3–5 cm), P3 (5–10 cm), P4 (10–15 cm), P5 (15–22 cm), and P6(22–30 cm)] and five stages of seed [S1 (0–2 DAP), S2 (3–4 DAP), S3 (4–10 DAP), S4 (11–20 DAP) and S5 (21–29 DAP)]. The colour scale at the bottom of each heat map is given in log2 intensity value. (TIF) [file pone.0062494.s002.tif]

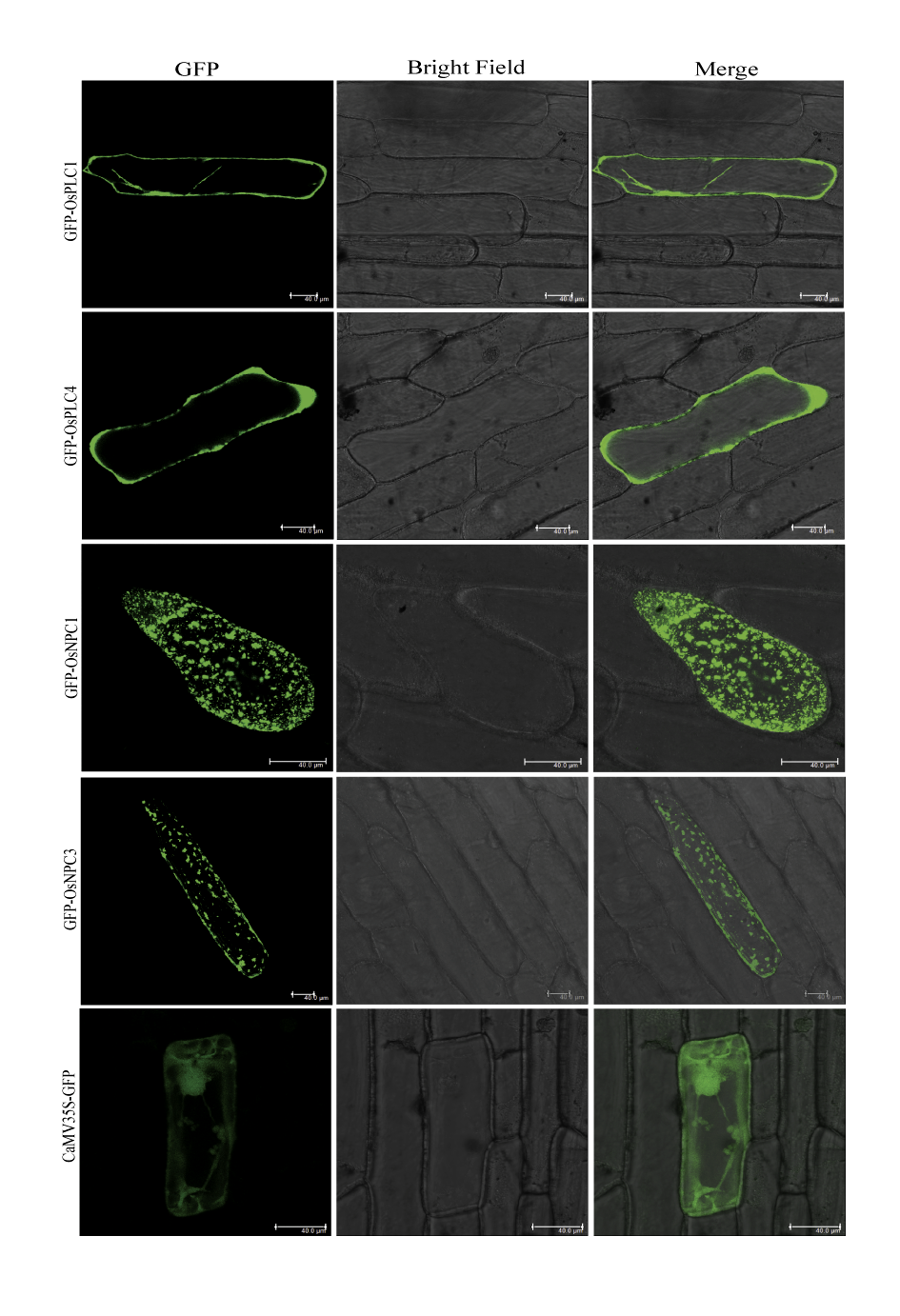

Supplement: Figure S3 — Subcellular localization of rice PLC proteins in onion epidermal cells. Onion epidermal cells expressing the GFP-PLC fusion protein driven by the 2X CaMV35S promoter. Confocal images of fluorescence (green) are shown for onion cell expressing GFP-OsPLC1 and GFP-OsPLC4 fusion protein showing its distribution throughout the cytoplasm and nucleus (upper two rows); expressed GFP-OsNPC1 and GFP-OsNPC3 fusion protein showing their preferential dotted localization (third and fourth rows). Cells transformed with vector only (CaMV35S-GFP) are shown in the lowermost row. All the images were taken in 5 different sections in z direction and merges together. Scale bar = 40 µm. (TIF) [file pone.0062494.s003.tif]

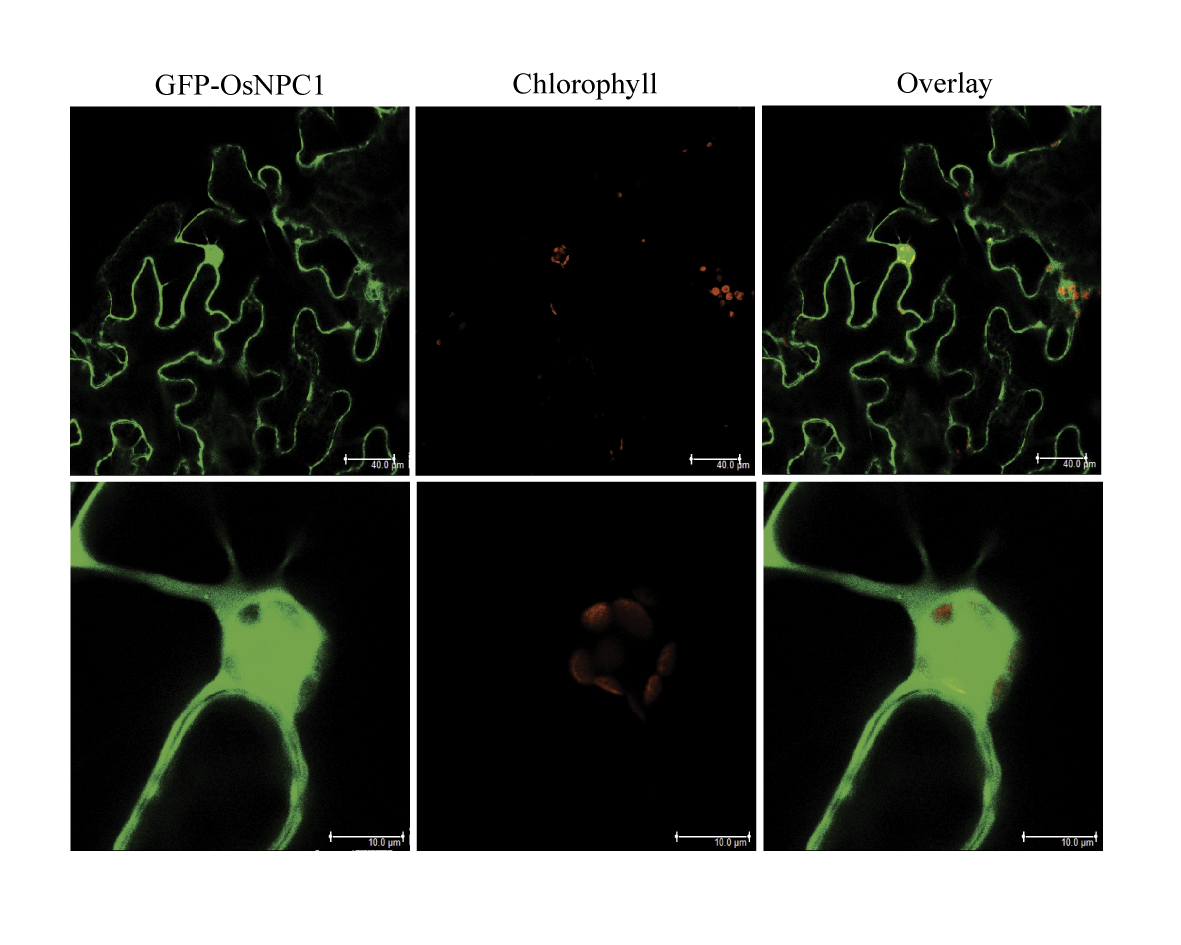

Supplement: Figure S4 — Preferential localization of OsNPC1 proteins in close proximity of chloroplast in Nicotiana cells. Expressed GFP-OsNPC1 fusion protein in Nicotiana cells, the upper panel showing cytoplasmic localization with small spots in the cell, which surround the chloroplasts in the overlay (Scale bar = 40 µm), which can be seen clearly in the magnified view of the spot in the lower panel. (Scale bar = 10 µm). All the images were taken in 5 different sections in z direction and merges together. (TIF) [file pone.0062494.s004.tif]
